# Lakeshore vegetation: More resilient towards human recreation than we think?

**DOI:** 10.1002/ece3.10268

**Published:** 2023-07-08

**Authors:** Nora Meyer, Anna Swiatloch, Sebastian Dittrich, Goddert von Oheimb

**Affiliations:** ^1^ Institute of General Ecology and Environmental Protection Technische Universität Dresden Tharandt Germany

**Keywords:** biodiversity, ecological impacts, freshwater, recreation ecology, shoreline vegetation

## Abstract

Lakes and their shoreline vegetation are rich in biodiversity and provide multiple functions and habitats for fauna and flora. Humans are attracted by the scenic beauty of these ecosystems and the possibilities for recreational activities they offer. However, the use of lakes for recreational activities can lead to disturbance of vegetation, threatening the integrity and functionality of shoreline areas. Recent literature reviews revealed that impacts of the seemingly harmless activities bathing and lingering on the shore on lakeshore vegetation are poorly understood. In this study, we analysed the effects of shoreline use connected with bathing on the structure, composition and diversity of lakeshore vegetation. Vegetation relevés were recorded in 10 bathing and 10 adjacent control sites in the nature park ‘Dahme‐Heideseen’ (Brandenburg, Germany). In addition visitor counts were performed. The species composition and the cover of herbaceous and shrub vegetation differed between bathing and control sites, but all sites had a high percentage of plant species not typical for the community. The vegetation parameters did not correlate with visitor counts. The results indicate that the present visitor intensity in the nature park does not impact the vegetation severely.

## Graphical abstract

The results of our study indicate an impact of the accumulated use at the lakeshore and sunbathing area on plant community composition. However, species diversity expressed through diversity indices generally showed no difference between impacted and non‐impacted sites. The bathing sites did have a different species composition than the control sites, however, these different species lead to a similar diversity and cover in the impacted areas.

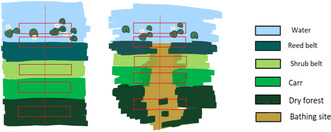

## 1 INTRODUCTION

Lakes have a particularly high biological diversity (Dudgeon, 2006). Because of their scenic beauty (Janeczko, 2009) and the opportunities for various activities they offer, lakes also represent a special attraction for visitors in a landscape. Lakes provide health‐promoting amenities associated with outdoor activities (Wolsko et al., 2019). In addition, lakes are rich in water resources, provide food and are used for hydropower (Schwarz, 2019) and transportation (Zajicek & Wolter, 2019).

**FIGURE 1:**
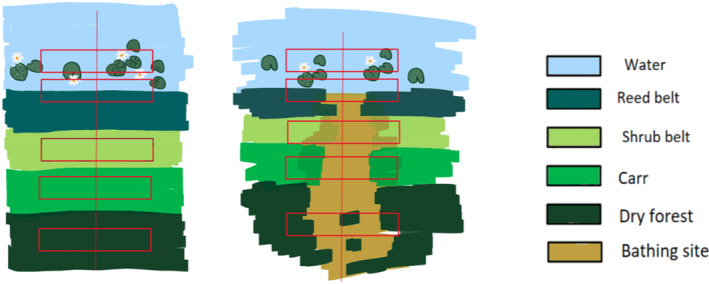
Schematic drawing of the study design. Bathing sites are shown on the right side, that for control sites on the left side. Red rectangles symbolize the positioning of the study plots. The colours represent the different vegetation zones.

The natural vegetation of lakes offers a variety of ecosystem functions (Meerhoff & de los Ángeles González‐Sagrario, 2021) and habitats in a relatively small space. This is particularly due to a small‐scale zonation based on shoreline relief and water depth, forming a gradient from wet, aquatic conditions and moist semi‐aquatic sites to mesic and dry sites higher above the lakeshore (Figure 1; Leuschner & Ellenberg, 2017). The lowest benthic sites are characterized by herbaceous aquatic vegetation that is either completely submerged or has floating parts, and completely floating species (*Potamogeton* spp., *Ranunculus* spp., *Nymphaeidae*, *Lemnaceae*; Chytrý, 2011). Such sites provide important feeding and reproduction habitats for both invertebrates and fishes (Vejříková et al., 2017). Intact bank vegetation dominated by reed belts is critical to the ecosystem, protecting the shoreline and providing food and shelter for numerous organisms (Meyer et al., 2021). Reeds such as *Phragmites australis* deliver various benefits to lakes, such as structural elements or habitat for highly specialized animal species (Ostendorp, 1993). Sites above the reed belt are often occupied by a shrub belt consisting mainly of willow species (*Salix* spp.). These shrubs can play the role of ‘keystone structures’ by providing habitats for insects (Noemí Mazía et al., 2006). This zone is followed by forest communities typical of waterlogged soils (*Alnus glutinosa*, *Betula* spp.) and finally by dry forests.

While the entire freshwater ecosystem is sensitive to climate change (Ormerod et al., 2010), the greatest threats to freshwater vegetation are eutrophication and streambed alteration (Leuschner & Ellenberg, 2017). However, tourism and recreation have been named as a threatening process for 42% of the critically endangered vascular plant species in Australia (Rankin et al., 2015). Shoreline‐related recreational activities such as walking, bathing and lingering on the shore can cause damage and death of individual plants, reduced vegetation cover and changes in species composition (Meyer et al., 2021), as well as degradation of lakeshores (Ostendorp, 1995a). Therefore, tourism and recreational activities must be recognized as an increasing threat to both lakeshore vegetation and the ecosystem services it provides (Chytrý, 2011; Rankin et al., 2015).

Recreational activities in forest and marine systems are relatively well studied (Larson et al., 2019), while freshwater ecosystems are generally underrepresented in biodiversity research and conservation (van Rees et al., 2021). Recent literature reviews (Meyer et al., 2021; Schafft et al., 2021) have revealed a gap in knowledge regarding how recreational activities affect the environmental quality and conservation objective of freshwater ecosystems. However, there is evidence of the negative impacts of recreational activities (Schafft et al., 2021). Focusing on the ecological impacts of shore‐based activities on plants, it becomes apparent that the number of studies on plants and vegetation is small, especially compared to studies on birds or fish (Meyer et al., 2021). The few studies that do exist suggest that activities could have lasting effects on plants under certain conditions, such as reduction in abundance and diversity and changes in species composition (Andrés‐Abellán et al., 2005; Bonanno et al., 1998; Bowles & Maun, 1982; Gremmen et al., 2003).

While the effects of walking and associated trampling on various ecosystems such as costal dunes (Andersen, 1995) or montane heathlands (Bayfield, 1979), and on various taxa such as insects (Ciach et al., 2017), aquatic macroinvertebrates (Hardiman & Burgin, 2011) and plants (Bernhardt‐Römermann et al., 2011; Cole, 1995; Gremmen et al., 2003) are well known, the effects of bathing activities are not well studied (Schafft et al., 2021). Most studies on the ecological effects of bathing focus on the toxicity of sunscreen products, while there are few studies on the effects of shoreline or aquatic recreational activities (Horn & Pätzold, 1999; Ostendorp, 1995a; Schafft et al., 2021).

Since lakeshores are divided into successive zones from water to land, each with characteristic indicator plant species and structures, generalized analyses do not fully reflect the complexity of this habitat. We therefore adopted a differentiating study approach and considered the different lakeshore zones separately. The typical zonation of central European lakes, for which visitor impacts are to be expected and which is subject of this study, includes the open water with submerged and floating vegetation, the reed belt, the moist shrub belt, the carr and the dry forest. The extent and presence of each zone is highly individual for each lake and varies according to lake bed morphology and trophic status (Pott & Remy, 2008). A detailed definition of the zones included in this study is given in the method section.

The vegetation of each zone could suffer from several impacts caused by humans engaging in recreational activities. The floating and submerged plants in close proximity to the shoreline are susceptible to damage from swimming and splashing. The reed belt, the moist shrub belt and the carr zone can be severely impacted by bathers as they have to move through these zones to get to the water and physical pressure is exerted when lingering on the shore. This might result in reduced vegetation cover and altered plant species composition, with fewer sensitive plant species than in the unimpacted shoreline areas. Yet, the impact could be limited to the shoreline and not extend to the adjacent forest areas. To fill the knowledge gaps mentioned above, we conducted a vegetation study on natural lakes with recreational use in Brandenburg, Germany. In the Dahme‐Heideseen Nature Park with special focus on recreation, we had the opportunity to study several lakes with similar ecological conditions, examining sections with both high and low impact of recreational activities. In this way, we aimed to test the hypotheses: (1) The lakeshores of bathing sites and unimpacted sites differ in terms of vegetation cover, plant community composition and plant species diversity; and (2) vegetation cover, plant species diversity and species composition are negatively correlated with visitation intensity.

## 2 METHODS

### 2.1 Study area

The study took place at 10 bathing sites in the Dahme‐Heideseen Nature Park, which is located 30–40 km south of Berlin, Germany. It belongs to the IUCN category ‘protected landscapes’, which has the goal to maintain a balanced interaction of nature and culture (Dudley, 2013). The nature park contains many lakes and is a popular destination for day or weekend trips, which makes it an ideal area for studying the impact of bathing activities on lakeshore vegetation. The area is located in the temperate climate zone with a mean annual temperature of 9.2°C and a mean annual precipitation of 576.0 mm (DWD, 2020). Sand was deposited in the study area during the Weichselian ice age, so that the soils today are dominated by nutrient‐poor, acidic sandy substrate (Stackebrandt & Franke, 2015).

To ensure maximum similarity, lakes surrounded by Scots pine (*Pinus sylvestris*) plantations and with similar trophic levels were selected for this study. The lakes are meso‐ to eutrophic with very clear water. There is a gradient of use in the study area ranging from large and easily accessible to small and hidden bathing sites. We selected sites that reflected the range of that gradient. Motor boating is prohibited on the selected lakes. Each bathing site was paired with a control site on the same lake. The control sites were located in close proximity to the bathing site, with similar orientation to the sun, but with no signs of human impact on the vegetation or terrain (such as tree planting or maintenance activities). Where multiple bathing sites were present at a lake, the one that was largest and most easily accessible from the parking lot was selected for this study.

### 2.2 Field work

To capture the variety of lakeshore vegetation, we established a study plot in each zone of natural vegetation zonation (Figure 1). The zonation in Table 1 is simplified after Hofmann and Pommer (2005) and Pott (1983) and represents the sequence of plant communities formed on the lakeshores in the study area in the absence of disturbance. Consequently, these zones are only developed in a near‐natural form at the control sites, while they deviate from this to varying degrees at the bathing sites. In each of the five zones, a rectangular plot with the size 2 m × 8 m = 16 m^2^ was established. The plot size was based on the recommendations of Chytry and Otýpková (2003). The position of the plot in each zone was determined by the presence of zone‐specific plant species rather than by a fixed distance from the water or by the vertical distance above lake level. This allowed to take the heterogeneity of the study sites into account. Plots were laid out parallel to each other in a transect from the water landwards (Figure 1). The plot containing the floating and submerged vegetation was placed at approximately 2 m away from the edge of the reed belt. The target vegetation communities for this zone were *Potamogetonetea* and *Lemnetea* (Table 1). The reed belt plot was located directly at the waterline, where *Phragmites australis* or *Typha* spp. grow in a dense belt, with the shorter side of the rectangular plot extending 1 m into the water and 1 m landward. The longer side of the plot was aligned parallel to the shoreline. The plot covering the moist shrub belt was established where the first willows (in particular *Salix fragilis*) and other shrub species grew. The first appearance of silver birch (*Betula pendula*) and common alder (*Alnus glutinosa*) determined the location of the plot in the carr zone, and Scots pine and various oak species (*Quercus petraea*, *Q. robur*, *Q. rubra*) were crucial for the positioning of the dry forest plots. The linear arrangement of plots was kept as a strict design, even at those bathing sites where bathing activities resulted in an absence of vegetation in the sample plot. If no vegetation grew in the plot, it was recorded as empty. If the vegetation did not contain the plant species typical of the zone, but the function was similar, the plot was placed there. In the following, the plots of the bathing sites are referred to as ‘impacted plots’, and those of the control sites as ‘control plots’.

**TABLE 1:** Assignment of the vegetation zones surveyed to units of the potential natural vegetation.

Zone	Target vegetation classes
Submerged and floating	*Potamogetonetea*, *Lemnetea*
Reed belt	*Phragmitetea australis*
Moist shrub belt	*Salicetea purpureae*, *Franguletea alni*
Carr	*Alnetea glutinosae*, *Alno‐Populetea*
Dry forest	*Quercetea robori‐petraeae* (Metzing, 2018)

Fieldwork was performed from the middle of June to the beginning of July 2020. In each plot, the overall plant cover as well as the cover of each plant species in the tree, shrub, herbaceous and moss layer was estimated in 5% intervals with a refinement at cover values <10% and <1% according to Dittrich et al. (2013). The vegetation layers are defined as follows: the tree layer was defined to be comprised of trees with a height of more than 7 m, the shrub layer includes woody species >1.5 and 7 m and the herbaceous layer included all vascular plants <1.5 m (including woody plants). The moss layer included all bryophytes and lichens growing in contact with the soil. Visitor counts were conducted every second weekend from the mid‐June through the end of August. Visitors to each bathing site were counted at different times during the weekend. Counts were performed on Fridays from 5 to 8 p.m. and on Saturdays and Sundays in three time slots from 11 a.m. to 5 p.m. The maximum number of visitors was determined for each bathing site and each day, and then the mean maximum number of visitors was calculated for each lake.

### 2.3 Data analysis

As a proxy for measured values on ecological site conditions, mean Ellenberg indicator values (EIVs) were calculated based on the vegetation relevés. They were expressed as cover‐weighted mean of the plant species recorded on each plot. EIVs for individual plant species were based on Ellenberg and Leuschner (2010), while nitrogen values for bryophytes were taken from Simmel et al. (2021). For definitions of the EIVs, see Table A1.

Based on the potential natural vegetation for each zone (Hofmann & Pommer, 2005), target vegetation classes were defined according to Berg et al. (2004). Phytosociological nomenclature follows Bergmeier (2020). Additionally to Berg et al. (2004), affiliation of single plant species to these classes were taken from Dierssen (2001), Oberdorfer (2001) and Berg and Dengler (2005).

The following further information was taken from the literature for each plant species:

1. conservation status (Caspari et al., 2018; Metzing, 2018)
2. naturalization status
3. growth form (Pigott & Ellenberg, 1984)

For each vegetation layer in each plot, the number of species, cover, ruderalization index, Shannon diversity index and evenness were calculated. The ruderalization index was calculated as the percentage share of non‐target species in total species and was inspired by the qualitative ruderalization index of Stroh et al. (2002). This index was calculated to determine how much the vegetation surveyed deviated from the potential natural vegetation. Plant species belonging to the target communities (see Table 1) are named target species, while species of any other community were classified as non‐target species. If a species could be assigned to two communities, both had to be counted in the respective analyses (Dittrich et al., 2016). The Shannon diversity index was calculated as

$$H=-\sum \frac{n_{i}}{N}\cdot \ln\left(\frac{n_{i}}{N}\right)$$

with *N* as the total number of species and *n*_*i*_ as the per cent cover of the *i*th species (Allaby, 2010). The evenness was calculated as: $E=H/H_{\max}$ and $H_{\max}=\ln\left(N\right).$

Differences in structure, composition and diversity between the impacted and control plots were examined using the pairwise Wilcoxon test. A PCA was performed on the vegetation relevés data. Analysis of group similarities (ANOSIM) from the vegan package (Oksanen et al., 2022) using the Bray–Curtis index of dissimilarity was used to quantify differences in species composition of the shrub, herb and moss layers between the impacted and control plots, also referred to as groups in this context further. The relationship between vegetation structure, community composition and diversity parameters and visitor intensity at bathing sites was analysed using Spearman rank correlations. All analyses were performed in R Core Team (2021). All figures were created using the package ggplot2 in the R environment.

## 3 RESULTS

A total of 134 plant species was found in the 10 bathing sites and 10 control sites. The most common tree species were *Betula pendula*, *Alnus glutinosa* and *Pinus sylvesteris*. *Phragmites australis* was the overall most common plant species, not only growing in the reed belt but also in the moist shrub belt. Other herbaceous species that were recorded frequently include *Deschampsia flexuosa* and *Lysimachia vulgaris*. The most common bryophyte species was *Mnium hornum*.

Both the submerged and floating vegetation and the moist shrub belt were present in only one impacted and two control plots, respectively. Since this does not represent a sufficient number of replicate samples, these two zones were excluded from further analyses. The indicator values showed that there were no significant differences between impacted and control plots within the zones (see Appendix ‘Zeigerwerte_Übersicht.xlsx’).

### 3.1 Reed belt

A belt of semi‐aquatic vegetation was present within the sample plots at seven bathing sites and nine control sites. The two vegetation layers tree layer and herbaceous layer were present (Table 2). Only one impacted plot, but six control plots had a tree layer. The herbaceous layer was formed in all vegetated plots of this zone, that is, *N* = 7 in impacted plots and *N* = 9 in control plots. The mean cover was significantly lower in both layers in the impacted plots (tree layer: mean = 1.5, SD = 4.7; herb layer: mean = 7.1, SD = 11.8) than in the control plots (tree layer: mean = 19.5, SD = 22.0; herb layer: mean = 34.5, SD = 19.9) (Table 3).

**TABLE 2:** Summary of the number of layers on the plots in the four zones studied.

Zone	Layer	Impacted plots	Layer	Control plots
Reed belt	Tree	1	Tree	6
	Shrub	0	Shrub	0
	Herbaceous	7	Herbaceous	9
	Moss	0	Moss	0
Moist shrub belt	Tree	1	Tree	2
	Shrub	1	Shrub	2
	Herbaceous	1	Herbaceous	1
	Moss	0	Moss	0
Carr	Tree	8	Tree	10
	Shrub	2	Shrub	10
	Herbaceous	10	Herbaceous	10
	Moss	7	Moss	4
Dry forest	Tree	10	Tree	10
	Shrub	3	Shrub	8
	Herbaceous	10	Herbaceous	10
	Moss	8	Moss	7

*Note*: In each case, the layer was counted as ‘present’ if vegetation at the corresponding height stratum was present on the plot.

**TABLE 3:** Differences between the impacted and control plots in the tree, shrub, herbaceous and moss layer.

Zone	Layer	Parameter	Impacted plots	SD	Control plots	SD	Significance
			Mean values		Mean values		
Reed belt	Tree	Species number	0.30	0.95	1.10	0.99	.05
Reed belt	Tree	Cover	1.50	4.74	19.50	22.04	.02
Reed belt	Tree	Ruderalization index	100.00	NA	41.67	37.64	.29
Reed belt	Tree	H index	0.86	NA	0.42	0.28	.29
Reed belt	Tree	Evenness	0.17	NA	0.08	0.05	.29
Reed belt	Herb	Species number	1.10	1.10	2.90	3.03	.09
Reed belt	Herb	Cover	7.12	11.79	34.50	19.92	.01
Reed belt	Herb	Ruderalization index	4.76	12.60	11.70	21.56	.49
Reed belt	Herb	H index	0.28	0.48	0.50	0.50	.28
Reed belt	Herb	Evenness	0.05	0.09	0.10	0.10	.28
Carr	Tree	Species number	1.00	0.67	2.40	0.84	.00
Carr	Tree	Cover	32.20	30.98	42.20	20.35	.36
Carr	Tree	Ruderalization index	62.50	44.32	45.00	29.45	.33
Carr	Tree	H index	0.16	0.30	0.56	0.34	.02
Carr	Tree	Evenness	0.03	0.06	0.11	0.07	.02
Carr	Shrub	Species number	0.50	1.27	2.10	1.52	.02
Carr	Shrub	Cover	1.60	3.50	17.70	24.07	.01
Carr	Shrub	Ruderalization index	30.00	42.43	27.08	26.80	1.00
Carr	Shrub	H index	0.54	0.76	0.69	0.51	.89
Carr	Shrub	Evenness	0.11	0.15	0.14	0.10	.89
Carr	Herb	Species number	11.40	6.15	7.50	4.28	.10
Carr	Herb	Cover	21.55	19.68	36.00	25.72	.15
Carr	Herb	Ruderalization index	71.20	14.70	39.77	26.52	.01
Carr	Herb	H index	1.52	0.55	1.15	0.73	.25
Carr	Herb	Evenness	0.30	0.11	0.23	0.14	.25
Carr	Moss	Species number	1.20	1.55	0.50	0.71	.34
Carr	Moss	Cover	0.86	1.59	0.42	0.93	.47
Carr	Moss	Ruderalization index	94.44	13.61	100.00	0.00	.54
Carr	Moss	H index	0.42	0.66	0.16	0.32	.69
Carr	Moss	Evenness	0.08	0.13	0.03	0.06	.69
Dry forest	Tree	Species number	1.60	0.84	2.30	0.82	.07
Dry forest	Tree	Cover	56.00	18.53	55.00	19.00	1.00
Dry forest	Tree	Ruderalization index	90.00	21.08	71.67	24.91	.10
Dry forest	Tree	H index	0.29	0.38	0.53	0.34	.14
Dry forest	Tree	Evenness	0.06	0.07	0.11	0.07	.14
Dry forest	Shrub	Species number	0.50	0.97	2.10	1.60	.02
Dry forest	Shrub	Cover	8.00	13.78	17.10	17.75	.12
Dry forest	Shrub	Ruderalization index	50.00	70.71	63.10	40.78	.88
Dry forest	Shrub	H index	0.30	0.52	0.59	0.47	.35
Dry forest	Shrub	Evenness	0.06	0.10	0.12	0.09	.35
Dry forest	Herb	Species number	4.90	4.20	7.30	3.02	.04
Dry forest	Herb	Cover	13.99	13.15	11.80	5.07	1.00
Dry forest	Herb	Ruderalization index	81.49	21.87	81.08	11.11	.67
Dry forest	Herb	H index	0.92	0.62	1.36	0.43	.06
Dry forest	Herb	Evenness	0.18	0.12	0.27	0.08	.06
Dry forest	Moss	Species number	1.60	1.26	1.20	1.14	.48
Dry forest	Moss	Cover	2.34	4.53	7.71	12.10	.85
Dry forest	Moss	Ruderalization index	100.00	0.00	91.67	14.43	.14
Dry forest	Moss	H index	0.35	0.42	0.22	0.28	.43
Dry forest	Moss	Evenness	0.07	0.08	0.04	0.06	.43

*Note*: *p*‐Values from Wilcoxon test. Significance < .05 >.

**FIGURE 2:**
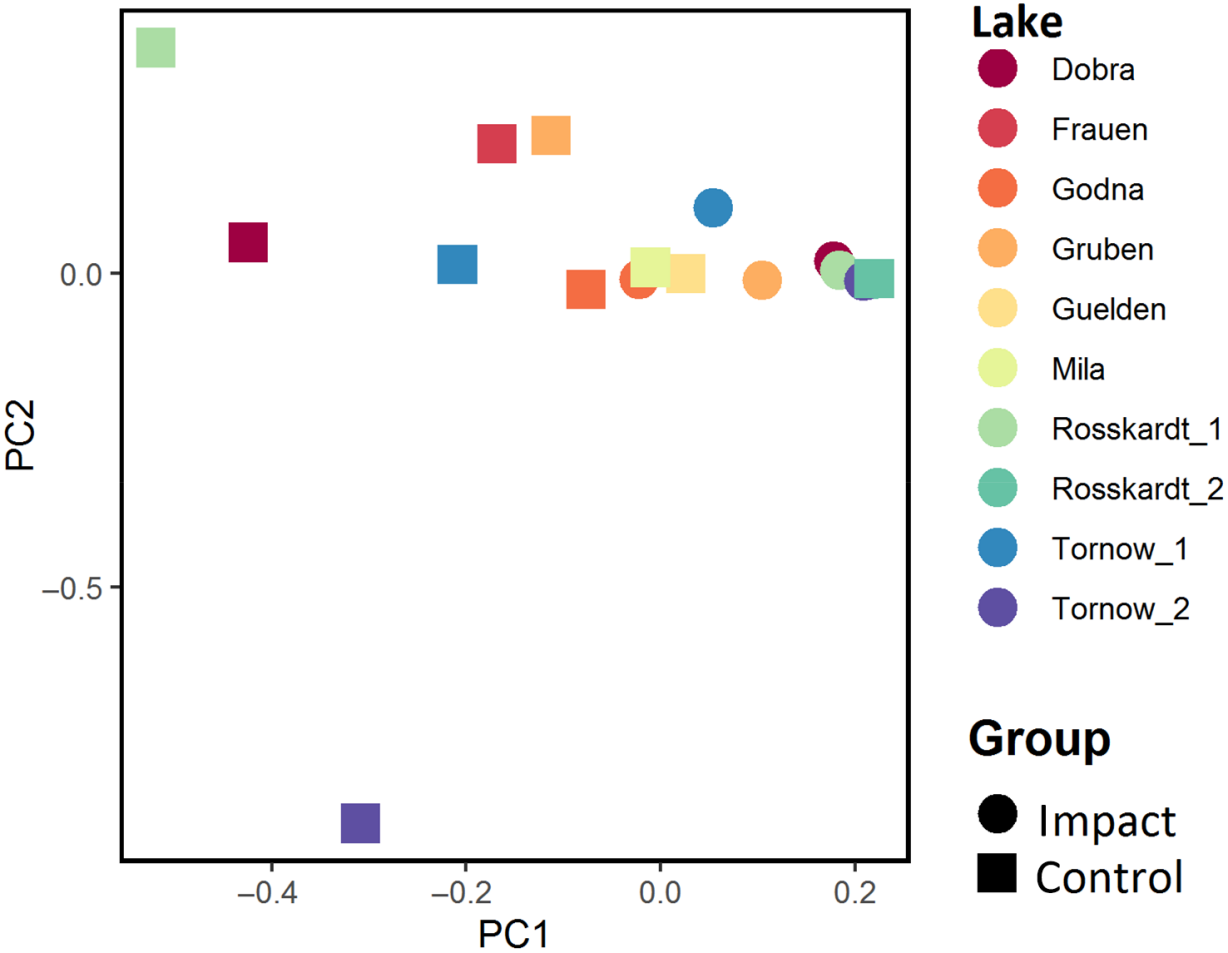
PCA of the reed belt zone. Colours refer to lakes, symbols to impacted plots (dot) and control plots (triangle).

The results of the ANOSIM led to a rejection of the null hypothesis that the similarity between groups is greater than or equal to the similarity within the groups (*p* = .004). The *R*‐value of .32 suggest a dissimilarity between impacted and control plots. This dissimilarity is also reflected in the PCA (Figure 2). The ruderalization index was lower in the herbaceous layer in the impacted plots (mean = 4.76, SD = 12.60) than in the control plots (mean = 11.70, SD = 21.56), but this difference was not statistically significant (*p* > .05) (Table 3). The herbaceous layer of the impacted plots (mean = 1.1, SD = 1.1) contained fewer species than the control plots (mean = 2.9, SD = 3.0), although this difference was not statistically significant. Likewise, the Shannon index and the evenness of the herbaceous layer did not show significant differences.

Among the three zones analysed, the reed belt was the zone with the highest number of significant differences in percent cover of life forms between the impacted and control plots (Tables 3 and 4). Specifically, we found significantly lower cover of geophytes, hydrophytes and phanerophytes in the impacted plots than in control plots.

**TABLE 4:** Significant differences of the cover (in %) of life forms between impacted and control plots.

Zone	Life form	Impacted plots	SD	Control plots	SD	Significance
		Mean cover		Mean cover		
Reed belt	Geophytes	4.02	6.26	29.41	20.94	.00
Reed belt	Phanerophytes	1.50	4.74	16.91	20.63	.01
Reed belt	Hydrophytes	6.62	10.18	34.95	20.56	.01
Carr	Phanerophytes	0.42	0.94	15.92	26.49	.01
Carr	Therophytes	0.70	1.25	0.03	0.09	.02

*Note*: *p*‐Value from Wilcoxon test.

### 3.2 Carr zone

The carr zone was formed in all bathing sites and control sites. While the tree and herbaceous layer was present in almost all plots, there were differences in the shrub and moss layer (Table 2). A shrub layer was formed in only two of the impacted plots, while it was present in all control plots. A moss layer was present in seven impacted and four control plots. Vegetation cover in the shrub layer was significantly lower in the impacted plots (mean = 1.6, SD = 3.5) than in the control (mean = 17.7, SD = 24.1) plots (Table 3). In the tree and herbaceous layer the cover was lower in impacted (mean = 32.2 and 21.6 respectively) than control plots (mean = 42.2 and 36.0 respectively), but differences were not significant.

**FIGURE 3:**
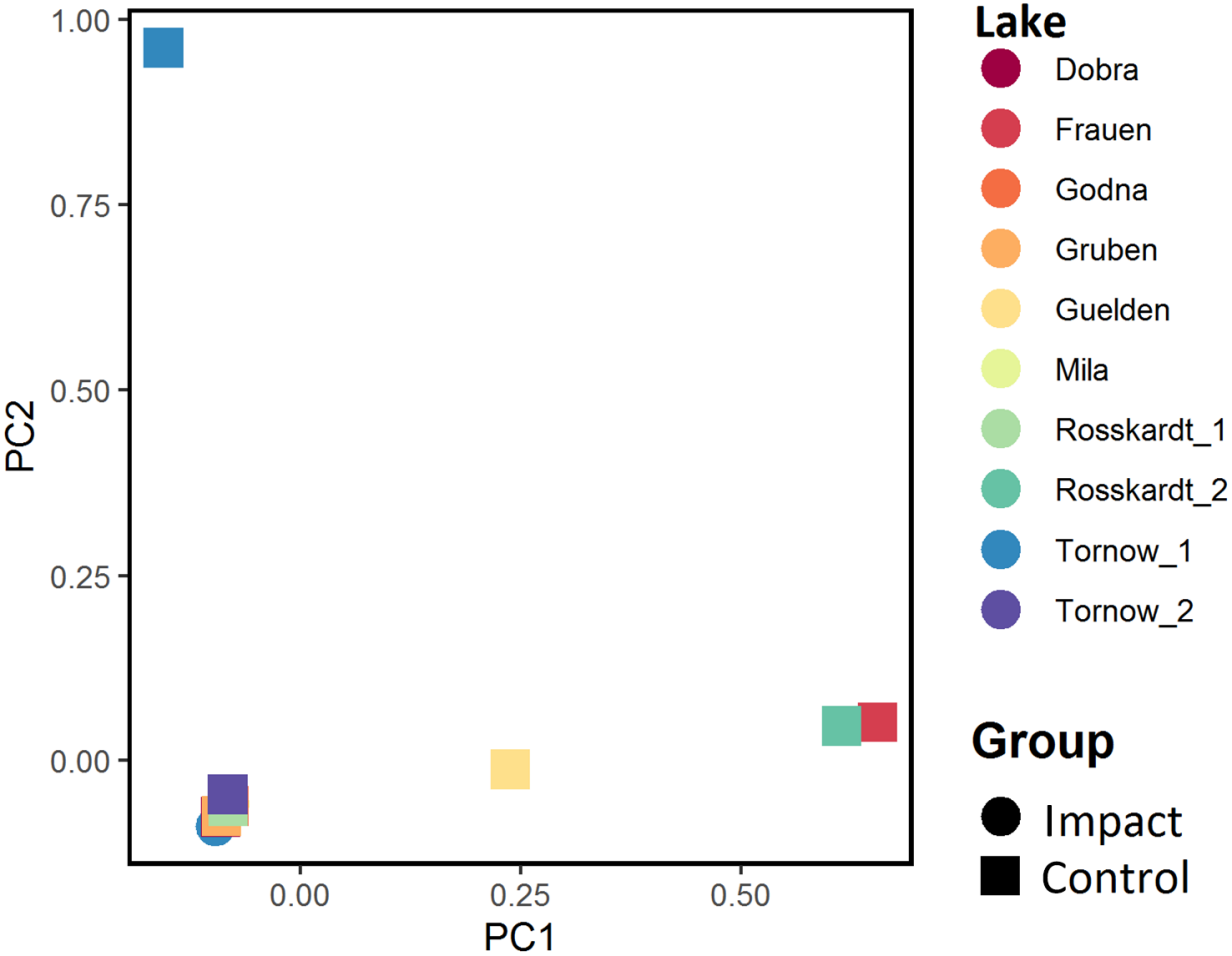
PCA of the carr zone. Colours refer to lakes, symbols to impacted plots (dot) and control plots (triangle).

The ANOSIM suggests a significant difference of species composition between groups in the carr zone (*p* = .001, *R* = .28). This is also reflected in the PCA (Figure 3), in which the impacted plots are aggregated while the control plots are more dispersed.

The ruderalization index was significantly higher in the herbaceous layer of the impacted plots (mean = 71.2, SD = 14.7) than in the control plots (mean = 39.8, SD = 26.5) (Table 3). No significant differences of the ruderalization indices were found in the other layers.

Species numbers in the tree and shrub layers were significantly lower in the impacted plots than the ones in the control plots (Table 3). The number of species in the herbaceous layer varied greatly between the impacted and control plots (mean = 11.4, SD = 6.2 and mean = 7.5, SD = 4.4 respectively). Therefore, despite relatively large differences in mean values, no significant differences were detected. Shannon index and evenness of the tree layer were significantly lower in the impacted plots than in the control plots. In six of eight impacted plots, the tree layer consisted of only one species. In contrast, nine of 10 control plots had two or more tree species. In two plots, even four tree species were found.

Cover of phanerophyte species was significantly lower in the impacted plots than in control plots, due not only to lower cover in the tree layer, but also to a lower presence of seedlings and regeneration (Table 4). Therophyte cover was significantly higher in the impacted plots than in the control plots.

### 3.3 Dry forest

The dry forest zone was present at all bathing and control sites. Within this zone, the tree layer and the herbaceous layer existed in all plots (Table 2). There were fewer plots with a shrub layer in the impacted plots (*N* = 3) than in the control plots (*N* = 8). The moss layer was present in eight impacted and seven control plots. No significant differences were found in the vegetation cover of the impacted and control plots (Table 3).

**FIGURE 4:**
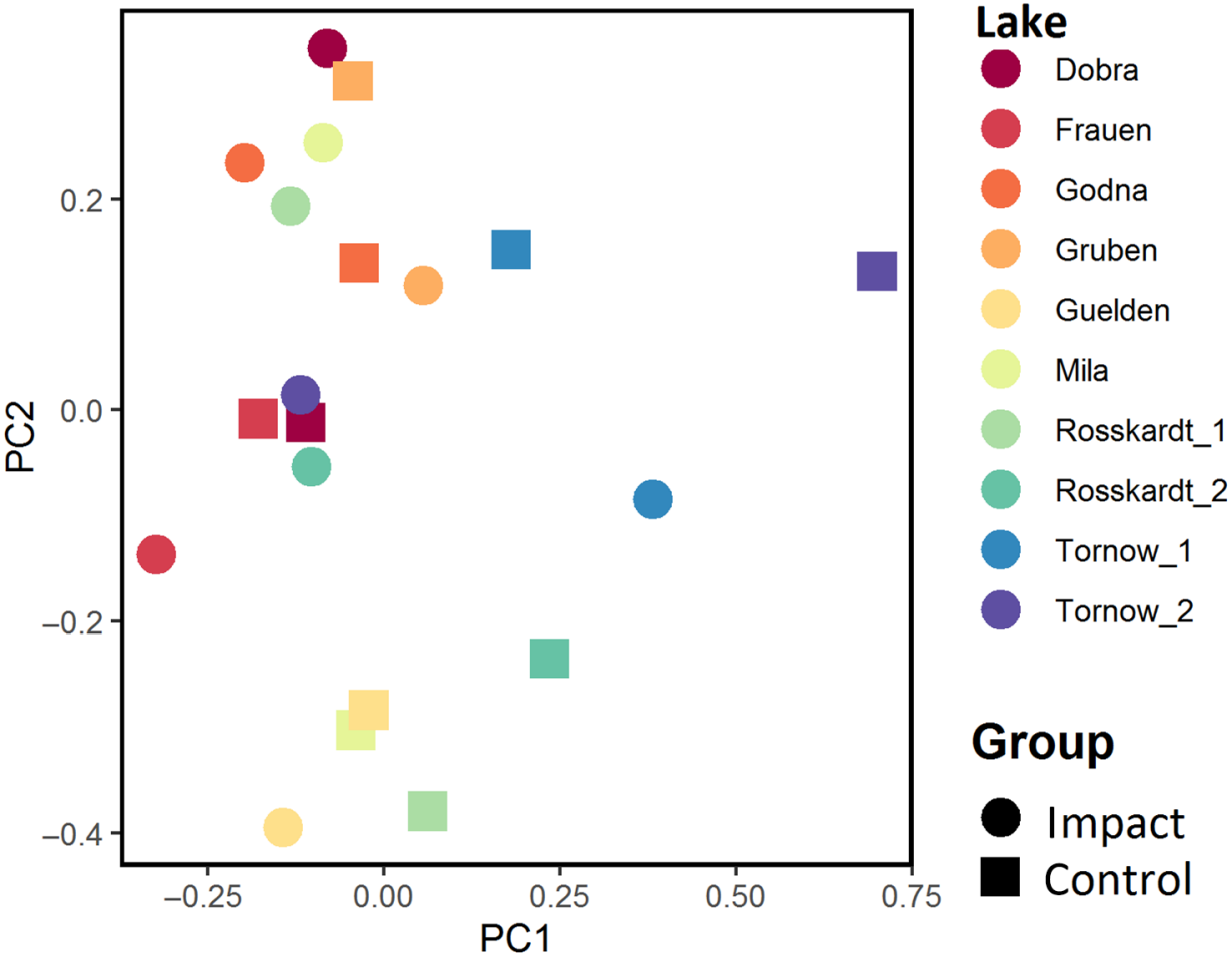
PCA of the dry forest zone. Colours refer to lakes, symbols to impacted plots (dot) and control plots (triangle).

Species composition of the dry forest zone was similar in impacted and control plots (*p* = .10 and *R*‐value = .07), indicating completely random grouping. This wide range of variability in species composition is also evident in the PCA (Figure 4).

Although the ruderalization index was higher in the tree and moss layer of the impacted plots than in the control plots, these differences were not statistically significant (Table 3). The tree layer in most of the impacted plots consisted of only one tree species (six of 10 plots), while in nine control plots at least two tree species and a maximum of four tree species formed this layer. In the impacted plots where a shrub layer was present, the number of woody species was low (three plots with only one woody species, one plot with three woody species). In contrast, the shrub layer of the control plots (with one exception) always contained more than one woody species. The herbaceous layer was significantly less species‐rich in the impacted plots (mean = 4.9, SD = 4.20) than in the control plots (mean = 7.3, SD = 3.02). The moss layer, when present, was species poor and consisted of one to three species, with the exception of one plot that had four species. No significant differences in the Shannon index and in the evenness of impacted and control plots occurred in any layer.

### 3.4 Impact of visitor intensity on vegetation

In the carr zone, the ruderalization index in the herbaceous layer and moss layer was negatively correlated with visitor intensity (Table 5). In the dry forest zone, the Shannon index and the evenness of the moss layer were negatively correlated with visitor intensity. Species composition of the reed belt, the carr zone and the dry forest was not affected by visitor intensity. The correlation coefficients of these three zones between the community composition variable and the visitor numbers were low (*ρ* ≈ .1) and the correlations were not significant (*p* > .05).

**TABLE 5:** Spearman rank correlation of parameters with visitor intensity.

Zone	Parameter	Significance	Rho
Carr	Ruderalization index in the herbaceous layer	.04	−.58
Carr	Ruderalization index in the moss layer	.04	−.71
Dry forest	H index of the moss layer	.01	−.77
Dry forest	Evenness of the moss layer	.01	−.77

*Note*: *p*‐Values from Spearman rank correlation.

A significant positive correlation was found between visitor intensity and the cover of *Holcus mollis* in the carr zone and that of *Hypochaeris radicata* in the dry forest zone (Table 6). In contrast, the cover of *Molinia caerulea* and *Agrostis stolonifera* decreased significantly with visitor intensity in the carr zone. *Hieracium pilosella*, *Poa palustris*, *Rumex hydrolapathum*, *Scutellaria galericulata* and *Trifolium campestre* had a positive but non‐significant correlation with visitor intensity in the carr zone (*ρ* > .5). *Holcus mollis*, *Calluna vulgaris*, *Vaccinium vitis‐idaea* in the dry forest and *Cerastium holosteoides* in the carr zone were negatively correlated with visitor intensity (*ρ* < −.5), but these correlations were not statistically significant. There was no significant correlation between the percentage of cover of the different life forms and visitor intensity.

**TABLE 6:** Correlation between plant cover of individual species and visitor intensity.

Species	Zone	Significance	Rho
*Agrostis stolonifera*	Carr	.03	−.68
*Holcus mollis*	Carr	.02	.70
*Hypochaeris radicata*	Dry forest	.03	.68
*Molinia caerulea*	Carr	.03	−.68

*Note*: *p*‐Values from Spearman rank correlation.

## 4 DISCUSSION

The results of this study revealed mixed support for the hypothesis that lakeshore vegetation differs in terms of plant cover, community composition and species diversity due to bathing activities. Bathing sites generally showed impoverished vertical structures, a lower cover and a changed plant species composition compared to the control sites. The impacted plots frequently lacked a tree or shrub layer, and the cover values of all vegetation layers were reduced. Species composition differed between bathing and control sites. However, no strong impact of the bathing activities on the diversity patterns was detected. However, in accordance with our second hypothesis, the diversity correlated negatively with visitor numbers in the herb and shrub layer of the carr zone. In the dry forest, the evenness and the Shannon index of the moss layer correlated negatively with visitor numbers. Besides these general findings it turned out that, the three zones that were included in this study showed different responses: the reed belt and the carr zone were much more affected than the dry forest. This confirms our decision to adopt a differentiated study approach, in which the various lakeshore zones were considered separately.

Based on our study no statements can be made about the submerged and floating vegetation, because in most sites no plants were found in this zone. Even though one study exists that links the decline of aquatic macrophyte diversity and cover with recreational activities in lakes (Cragg et al., 1980), in our case the absence of macrophytes cannot directly be attributed to the effects of human recreation. Other factors such as water level fluctuations or nutrient levels might be accountable for the lack of plant cover in this zone. Assuming that swimmers tend to avoid lakes with dense underwater vegetation as it hinders underwater movement, it is also possible that the lakes chosen for this study naturally contain only few macrophytes as we selected only lakes at which recreational activities are present.

Beforehand, the reed belt was presumed to be a hotspot of anthropogenic pressure and the results show that the hypothesis that bathing sites and control sites differ in terms of vegetation cover and species composition can be confirmed for the reed belt. Differences in cover can be related to the opening in the reed belt for entering the water. The mean cover of the impacted sites is nearly 25% lower than that of the control sites. This seems to be a serious impact on the shoreline and its vegetation and agrees with findings from Ostendorp (1995b), who identified recreational activities as one of the main causes for lakeshore deterioration and reed decline. The reed belt has a very high structural and functional significance for the shoreline by protecting its structure and providing a habitat for multiple organism groups (Meyer et al., 2021; Ostendorp, 1993). The reed belt itself is generally a species poor community and the bathing activities seem to lead to an even lower diversity in this zone. This difference was even reflected in the significantly different species composition of control and impact reed belt plots. However, in term of the entirety of the lakeshore, this impact appears minor provided that the majority of the shoreline is free from human intervention. This is the case at the lakes studied.

The vegetation of the carr zone also strongly reacted to the impact of bathing activities. The diversity of trees was significantly lower in impact plots, which suggest that not all of the tree species present in the area were able to grow in these highly disturbed habitats. The cover of shrubs was significantly lower on impacted plots and the species composition differed significantly between groups. Nearly all control plots contained woody species being present in this height stratum while nearly none were present in the impacted plots. This suggests that the repeated disturbance severely impacts the growth and survival of woody species in the shrub layer. As shrubs play an important role by providing shelter and food for wildlife such as birds (Jacobs et al., 2012) and catalysing succession (Gómez‐Aparicio, 2009), this is problematic for the shoreline ecosystem.

The herbaceous layer of the carr zone is directly affected by the activities trampling and sunbathing. However, contrary to our expectations, we did not find significant differences in cover or species numbers. This is contrary to other studies focussed on vegetation close to water bodies, where impacted sites had a reduced cover and species richness compared to control sites (Andrés‐Abellán et al., 2005; Bonanno et al., 1998). Our results rather agree with findings from Herben et al. (2018) who concluded that even low productive habitats can support plant life on frequently disturbed grounds when the disturbance is not severe. Another explanation for the lack of differences between the herbaceous layer in control and impact plots may be that there is a lower light availability in most control plots due to the more dense canopy cover. Marion et al. (2016) stated that the amount of sunlight was the most influential predictor of vegetation cover. Therefore, the lower light availability could hinder the growth of herbaceous vegetation and mask the differences caused by trampling in the impact plots.

Significant differences in species compositions of control and impact plots were found in the carr zone. One aspect leading to this difference is the significantly higher percentage of ruderal species in the herbaceous layer of impacted plots. This was caused by an increased presence of resistant, non‐target species with the decrease in fragile target species over time (Sun & Liddle, 1993b). Trampling resistant species such as *Lollium perenne* grew in the sunbathing area, also leading to a similar herb cover in impacted and non‐impacted sites. As mentioned above, the difference in light availability is also a factor contribution to the difference in species composition.

There were significantly more therophytes in the impacted plots than in the control plots of the carr zone. The strategy of recovering from the seeds seems to be an advantage in the trampled areas where plant parts of, for example hemicryptophytes, would be destroyed by frequent physical disturbance. Many species of highly disturbed sites are short‐lived annual species with high fecundity and a persistent seed bank (Rees & Long, 1992), while long‐lived and woody species are widely displaced from such sites, and may re‐increase after anthropogenic disturbances cease (Brandes, 2007).

Beforehand, we did not expect to observe significant differences between impacted and control plots of the dry forest zone. Because the sunbathing area of the bathing sites did not conflict with this zone in most cases, they were not as intensively trampled as the carr zone and the reed belt. There was also very little littering observed. The differences between control and impact plots in the dry forest zone were less pronounced than in the other zones. Despite lower species numbers in the shrub and herbaceous layer in the dry forest, no difference in diversity or ruderalization indices was found. The shrub layer had significantly less cover in impacted forest plots, which cannot be correlated with differences in silvicultural management. In forest, lower vegetation such as herbs and the emergence of shrubs is quickly lost even under low levels of traffic (Marion et al., 2016), which might explain the differences in cover and species numbers in the dry forest zone even though the pressure of visitors was low. Evidence suggest that woody species are at a disadvantage in resisting trampling (Sun & Liddle, 1993a) which could explain why the shrub layer is reduced not only in the dry forest zone but also in the carr zone.

The moist shrub belt was not present in most impacted and control sites. This shows that not the recreational use is responsible for the lack of the moist shrub belt. This might be due to the morphological structure of the shoreline. In particular, the embankment might be too steep for evolving a transitional zone between the carr zone and the reed belt. In such cases, shrubs might be displaced by shading from the adjacent forest, which is also constitutes the subsequent stage in lakeshore succession (Weber, 1998).

The hypothesis that visitor intensity would negatively correlate with plant community composition, species diversity or vegetation cover cannot be supported by our results. Only the diversity of the moss layer in the dry forest showed a significant negative correlation with the number of visitors. This is consistent with the findings of Studlar (1980) particularly for large forest and swamp bryophytes. There was also no correlation between the visitor intensity and the growth form of plants, which is contrary to the assumption that higher visitor intensities would lead to more robust growth forms.

The lack of correlation between visitor intensity and the parameters studied here may also be due to a small range of visitor intensity. Most bathing sites had a low to medium visitor pressure and only one bathing site was intensely visited. Studies with a wider spectrum of visitor pressure would be needed to find the maximum carrying capacity.

The analysis of visitor numbers in this study has shown that fragile species (*Molinia caerulea*, *Agrostis stolonifera*) are negatively correlated with visitor intensity. *Holcus mollis* and *Hypochaeris radicata* were positively correlated, growing even in highly frequented sites. The increase in cover of *H. mollis* and *H. radicata* might be due to higher light availability at larger and thereby less shadowed and more intensively used bathing sites. *H. radicata* is also known to form rosettes and benefit from moderate trampling (Oberdorfer, 2001). *M. caerulea* decreased with rising visitor numbers, which might be due to its need for permeable soil.

An explanation for the missing correlation between the community composition, visitor intensity and diversity, cover or growth form might be that the visitor numbers recorded here were comparably low. The area contains many lakes and bathing sites so that visitors can spread out and avoid crowded places. Another explanation might be that the area is already so intensively modified by centuries of anthropogenic use so that the lakes are already equally altered and the additional pressure through bathing activities does not have a great impact.

## 5 CONCLUSION

In conclusion, the results of our study indicate an impact of the accumulated use at the lakeshore and sunbathing area on plant community composition. However, species diversity expressed through diversity indices generally showed no difference between impacted and non‐impacted sites. The vegetation cover did not differ in most sites. The bathing sites did have a different species composition than the control sites, however, these different species lead to a similar diversity and cover in the impacted areas. As even the ruderality of impacted and control sites is similar, the ecological effects of the bathing activities observed in this study can be classified as low. The visitor intensity at the lakes studied here seems to be compatible with a more or less undisturbed vegetation. Only woody species such as shrubs were nearly entirely removed from sunbathing areas, but as they only presented a small section of the entire area, this effect is not regarded as a severe disturbance.

The low visitor intensity currently present in the Nature Park Dahme‐Heideseen is due to the multiplicity of easily accessible and suitable lakes for recreation. As long as visitor pressure on the shoreline vegetation does not exceed a threatening level and the proportion of shoreline sections used remains low, the vegetation and the ecological functions it provides are not at risk. The bathing activities in the Nature Park rather represent an example on how human needs for recreation can act in balance with nature. In regions with a higher proportion of used shoreline area and other shoreline uses, the additional pressure through bathing gains in importance. To determine at which intensity level vegetation gets heavily impacted, further studies are needed.

## AUTHOR CONTRIBUTIONS

**Nora Meyer:** Conceptualization (equal); data curation (lead); formal analysis (lead); methodology (equal); visualization (lead); writing – original draft (lead); writing – review and editing (equal). **Anna Swiatloch:** Data curation (equal); formal analysis (equal); visualization (equal); writing – review and editing (equal). **Sebastian Dittrich:** Conceptualization (equal); methodology (equal); supervision (equal); writing – review and editing (equal). **Goddert von Oheimb:** Conceptualization (equal); funding acquisition (lead); supervision (equal); writing – review and editing (equal).

## FUNDING INFORMATION

Nora Meyer received funding from the German Federal Ministry of Education and Research (Grant number 02WRM046).

## CONFLICT OF INTEREST STATEMENT

All authors declare that they have no conflict of interest.

## Data Availability

The data that support the findings of this study are available from the corresponding author upon reasonable request. Supplementary material can be found at https://zenodo.org/record/8043387.

## APPENDIX 1

**TABLE A1:** Definition of Ellenberg's indicator values (after Ellenberg & Leuschner, 2010; Simmel et al., 2021).

Class	L	R	M	C	T	N
1	Deep shade (<1)	Strongly acidic (<3.0)	Extremely dry	eu‐oceanic	Cold	Extremely infertile
3	Shade (<5)	Acidic (<5.0)	Dry	Widely Central European	Cool	Moderately infertile
5	Half shade	Moderately acidic (5.0–6.0)	Fresh	Weakly suboceanic to weakly subcontinental	Moderately warm	Moderately fertile
7	Semi‐light	Weakly acidic–moderately basic (6.0–6.9)	Moist	Subcontinental to continental	Warm	Richly fertile
9	Full sun (>50)	Basic, calcareous (≥7.0)	Constantly wet	Continental	Extremely warm	Overly nutrient rich

*Note*: L, light preference (per cent of relative irradiance), R, substrate reaction tolerance (pH value), C, continentality (based on natural distribution range); T, temperature optimum (gradient from lowland to mountainous, Arctic to Mediterranean), M, moisture preference, N, nutrient supply. Even classes, which represent transitions between the odd classes, were omitted.
